# Annotations

**Published:** 1910-01-29

**Authors:** 


### ANNOTATIONS.

### A Question of Ethics.

UNFORTUNATELY for the reputation of the profession in the eyes of the public, it is undeniably a fact that a great many of the offences, or alleged offences, against a somewhat mysterious unwritten code of medical ethics, which from time to time agitate the breasts of practitioners and are duly ventilated in the professional press, are trivial and even almost factious, if not factitious. That this is so must not, of course, prevent a prompt denunciation of anything which clearly and evidently oversteps the limits of decency and good taste. In no connection is this more important than in respect of advertisements in the professional press; for this is not only a question of good taste or square dealing on the part of the advertiser, but involves also the standing and reputation of a much more representative institution of the medical world, the journal itself. An advertisement has appeared lately in the columns of the official organ of the British Medical Association about which we will only say that its impropriety is obvious, and that we are amazed to see it in the place we have mentioned. Perhaps almost equally astonishing is the effrontery of the doctor by whom the advertisement is inserted. The notice in question runs thus: "A doctor practising in New Zealand can introduce medical men to PARTNERSHIPS, PRACTICES, and OPENINGS. Five per cent. commission on first year's income. Home references." Without doubt this advertisement has been inadvertently accepted, most probably without the editor's knowledge; but as the custodian of professional morals, and the very fountain-head of pure medical ethics, it behoves the *British Medical Journal* to be doubly careful in admitting anything to which exception can rightly be taken. The position of the Association when endeavouring to check abuses is not strengthened if members of the public—or black sheep within the fold—can point to such gross violations of the spirit of professional honour as are contained in every word of the paragraph quoted.

### Cold Storage of Vaccine Lymph.

THE report of the Medical Officer of the Local Government Board, which was issued this week, contains a report dealing with vaccine lymph kept in cold storage for periods of two years and six months respectively. Dr. Blaxall and Mr. Fremlin, who conducted the investigation, state that when the two-years-old lymph was withdrawn from cold store it was found to be free from extraneous organisms, and when used by public vaccinators in the vaccination of 8,559 cases gave "case" and "insertion" percentage successes of 97.8 and 91.4 respectively. The portions which had been exposed to a temperature below freezing point for six months were used for 40,931 vaccinations, and gave a case success of 99.6 per cent. and an insertion success of 96.7 per cent. The results obtained in the latter cases were practically identical with those obtained from lymph, issued to public vaccinators without cold storage six to eight weeks after collection. The Medical Officer says: "These results have considerable importance, since cold storage will enable a supply of lymph to be prepared and stored to meet any sudden expansion in the demand for lymph that may arise by reason of an outbreak of small-pox."

### Dangerous Hair Dressings.

A FEW months ago a death was caused by the use of carbon tetrachloride as a shampoo for the hair, and this was followed shortly afterwards by several fatalities due to the use of inflammable hair washes. The question of placing some restriction on the use of these substances is now under consideration by the proper authorities, but in the meantime definite steps have been taken in the United States of America to prohibit the sale of dangerous hair dressings. A Bill has been introduced in the House of Representatives which makes it an offence for any person to sell any substance for shampooing or cleaning the hair which is liable, by inhalation or by absorption through the skin, to produce poisonous effects, and forbids the use of any substance which contains carbon tetrachloride, gasolene, benzene, or benzole.

### A Glance at Medical History.

UPSTAIRS, in the bronze room of the British Museum, there is now an extremely interesting exhibition illustrating Greek and Roman domestic life. One section, some half-dozen cases that is to say, contains for the medical practitioner much to fascinate his interest. There is a delightful group of medical and surgical appliances used in the great days of Athens and Rome. There is, in the first place, a scene from a fifth century vase-painting, which shows a surgeon at work, bleeding a patient in the arm. At the iatreion, or surgery, "a dwarf slave is ushering in other patients, and bleeding cups are hanging on the wall." The surgical instruments, which are made chiefly of bronze, include a surgical saw, some forceps, one probably being an ovula forceps used for crushing the part intended to be amputated; spatulæ, eye-spoons for pouring lotions into eye, and many charming vases for unguents. The oculists' stamps are most interesting, containing, as they do, the information on their oblong surfaces of the oculist's name, the name of his specific, and its purpose. Surgery was, of course, a later development, owing to the difficulties attending the dissection of bodies, even the bodies of animals. When we remember that no degree or license to practise was necessary in Rome, as well as the ignorance of anatomy, if not of physiology also, we shall be inclined to agree with Pliny's words, quoted in the capital catalogue of the exhibits, that "the physician alone of men had liberty to kill." A visit to the present exhibition gives a vividness to our impressions of Græco-Roman medicine which few wise practitioners will be willing to lose.

